# Recombination events restored the functional horned haplotypes in the offspring of polled parents

**DOI:** 10.1186/s12711-025-01009-6

**Published:** 2025-10-31

**Authors:** Maulik Upadhyay, Alexander Graf, Neža Pogorevc, Doris Seichter, Ingolf Russ, Stefan Krebs, Saskia Meier, Ivica Medugorac

**Affiliations:** 1 Population Genomics Group, Department of Veterinary Sciences, LMU Munich, 82152 Martinsried, Germany; 2 SNSB-Genomics Core Facility, Menzinger Straße 67, 80638 Munich, Germany; 3 Laboratory for Functional Genome Analysis, Gene Center, Ludwig-Maximilians-University Munich, 80539 Munich, Germany; 4 Tierzuchtforschung e.V. München, 85586 Grub, Germany; 5 SYNETICS Germany GmbH, Research & Genetic Solutions Development, Osterkrug 20, 27283 Verden (Aller), Germany

## Abstract

**Background:**

Breeding of genetically polled animals is a desirable approach in modern cattle husbandry for welfare and economic reasons. At least four different genetic variants associated with polledness in cattle have been identified, suggesting genetic heterogeneity. These dominant variants are located on chromosome 1 between approximately 2.42–2.73 Mb (reference: ARS-UCD1.3), also called the *POLLED* locus. Among these variants, Friesian (*P*_*F*_, ~ 80 kbp duplication) and Celtic (*P*_*C*_, 212 bp complex InDel) are the most common across breeds in the production systems globally, such as in Holstein–Friesian (HF) and Fleckvieh (FV). While studies have provided strong evidence supporting the association of the *P*_*F*_ allele with the polledness, it has not yet been functionally validated, unlike the *P*_*C*_ allele.

**Results:**

Here, we conduct whole-genome sequencing analyses of two trios exhibiting unexpected inheritance patterns related to the *P*_*C*_ and *P*_*F*_ variants. In both instances, horned offspring were produced from mating pairs where one parent was homozygous for the polled variant and the other was homozygous for the ancestral horned variant. By analyzing the WGS data generated using Nanopore technology, we show that the de novo generation of the ancestral horned phenotype in both offspring was the result of distinct recombination events. Specifically, in the HF trio, it was the result of non-allelic homologous recombination in the gametes of the sire (*P*_*F*_*/P*_*F*_*)*, while in the FV trio, it was the result of allelic homologous recombination in the gametes of the dam (*P*_*C*_*/P*_*F*_). The findings from the HF trio support the hypothesis that ~ 80-kbp duplication is the genetic variant responsible for the polled phenotype of Friesian origin.

**Conclusion:**

We show that different genomic arrangements in the *POLLED* locus can lead to the emergence of de novo ancestral horn phenotypes. Such arrangements can complicate phenotype prediction in offspring, even when sires or dams have been tested as genetically homozygous polled. Therefore, it is important, for a better understanding of the relationship between the *POLLED* locus and the POLLED phenotype, that any deviation from the expected result is critically analysed. Possibly, some of these cases can further narrow down the sequence motif that is essential for polledness in cattle.

**Supplementary Information:**

The online version contains supplementary material available at 10.1186/s12711-025-01009-6.

## Background

Based on the size, shape, and characteristics of horns, the headgear phenotype in cattle can be categorized into: (i) true horns in bovids, consitsting in a pneumatized bony inner core that is attached to the frontal bone and have an outer keratin sheath [1]; (ii) scurs, that are rudimentary horn-like appendages that are loosely attached to the skin rather than the frontal bone with no pneumatized bony inner core; (iii) complete absence of horn-like appendages, a condition called polledness. Like their wild ancestors, the aurochs, and all members of the Bovidae family, cattle are naturally horned. Therefore, the presence of true horns is an ancestral characteristic of Bovidae, and scurs as well as polledness are derived traits. Horns have been associated with a diverse range of functions such as exerting territorial dominance, competing for mating and other resources, protection against predators, and maintaining thermoregulation [2–4]. The process of domestication and subsequent breed formation has largely rendered horns redundant. In fact, in modern industrial settings, breeding of genetically polled animals is a desirable approach for welfare and economic reasons [5]. To this end, a variety of chemical and surgical methods are used for disbudding or dehorning. Across the cattle sector, these practices are commonplace. All these procedures are labour-intensive and cause pain and distress to animals. Further, they also leave the wound sites exposed to infection, adding to the distress [5–7]. Therefore, the animal welfare guidelines recommend adapting husbandry systems to the ancestral characteristics of cattle or breeding genetically hornless animals rather than dehorning them.

Early and accurate detection of animals having a polled condition is a key to selective breeding for getting polled phenotype in a population [8]. Polled phenotype is a Mendelian trait governed by multiple variants (genetic heterogeneity) that are located on BTA1 (*Bos taurus* chromosome 1) between the region of approximately 2.42–2.73 Mb (reference: ARS-UCD1.3), also referred to as the *POLLED* locus throughout the manuscript. Phenotypic penetrance suggests that all these variants are dominant and cause polledness. Mainly, four polled variants have been identified. (i) Celtic (*P*_*C*_): This complex insertion-deletion variant, “g.[2429326_2429335del;2429109_2429320dupins]”, was first identified in several European breeds; since the geographical distribution of these breeds overlaps mainly with the area in which Celtic culture was practiced, it was named “Celtic” [9]. (ii) Friesian (*P*_*F*_): this tandem duplication variant, “g.2629116_2709243dup (80,128 bp)”, was first identified in Holstein–Friesian (HF) cattle [9, 10]. The duplicated segment has the same orientation as the reference sequence. It differs from the reference sequence by a SNP (single nucleotide polymorphism) (*P*_*T-*>*A*_*)* and a 2 bp deletion (*P*_*F2D*_*,* TG) at the 1st bp and 38th bp positions, respectively, on the duplicated sequence. (iii) Mongolian (*P*_*M*_): this complex insertion-deletion variant, “[2695261_2695267delinsTCTGAA;2695889_2696047dupins]”, was first identified in Mongolian Turano cattle and Mongolian yaks [11]. The *P*_*M*_ variant is located within the 80,128 bp segment duplicated by the *P*_*F*_ duplication. (iv) Guarani (*P*_*G*_): this duplication variant, “g.2614828_2724315dup”, was first identified in Nellore cattle from Brazil [12] and completely overlaps with *P*_*F*_ and *P*_*M*_. Interestingly, none of these four variants occur within coding regions. While several hypotheses have been proposed [13, 14], the exact pathways through which these complex variants disrupt the migration, proliferation, or differentiation of cells in the horn bud region remain unknown [15].

Among these four variants, *P*_*C*_ and *P*_*F*_ represent the most common polled variants observed across breeds worldwide. Of these variants, the causal association of *P*_*C*_ with polledness was proved by introducing the *P*_*C*_ allele from genome-edited cell lines, resulting in the birth of polled calves [16]. For the *P*_*F*_ variant, the presence of a large duplication (~ 80 kb) makes it difficult to prove the causality by genome editing or to pinpoint the exact genomic region responsible for the polledness. Efforts to further narrow down the region of the *P*_*F*_ variant, including analyses of recombination events in horned animals, have consistently identified the ~ 80 kb duplication as the sole variant associated with the *P*_*F*_ allele [10]. Notably, a study reported the absence of the diagnostic *P*_*F*_ SNP in a horned offspring from a trio in which the sire was homozygous for the *P*_*F*_ variant, further supporting the ~ 80 kb duplication as the only variant linked to the *P*_*F*_ allele [17]. Because the small *P*_*M*_ structural variant (SV) is embedded in the same region as the *P*_*F*_ allele, it could indicate that the more distal part of the 80 kb region is essential for polledness. Nevertheless, the question remains whether any SV along the entire *POLLED* locus (i.e., from the start of the *P*_*C*_ duplication (2,429,326 bp) to the end of the *P*_*G*_ segment (2,724,315 bp) is sufficient to disrupt the process of horn bud formation.

With the aim to better understand the underlying genetic mechanism of horn ontogenesis, we carried out a comprehensive bioinformatics analysis of two trios exhibiting unexpected inheritance patterns related to *P*_*C*_ and *P*_*F*_ variants. In both instances, horned offspring were produced from mating pairs where one parent was homozygous for the polled variant, and the other was homozygous for the ancestral horned variant. The polled gene test, targeting a 2-bp deletion (*P*_*F2D*_) and a 212-bp insertion (entire *P*_*C*_ variant), was used to confirm the presence of *P*_*F*_ and *P*_*C*_ variants, respectively. Specifically, in the HF trio, the sire was homozygous *P*_*F*_/*P*_*F*_, while in the Fleckvieh (FV) trio, the dam carried *P*_*C*_/*P*_*F*_ genotype. Therefore, the resulting offspring were expected to exhibit polledness by inheriting either the *P*_*C*_ or *P*_*F*_ polled variant. To our surprise, the investigated offspring from each trio showed the regular horn condition and *p*/*p* genotype instead. After confirming parentage with BovineSNP50 BeadChip, we hypothesized that recombination events in the *POLLED* locus of paternal germ cells in the HF trio and maternal germ cells in the FV trio may have led to novel genomic rearrangements, resulting in a variant resembling the wild type.

**Fig. 1:**
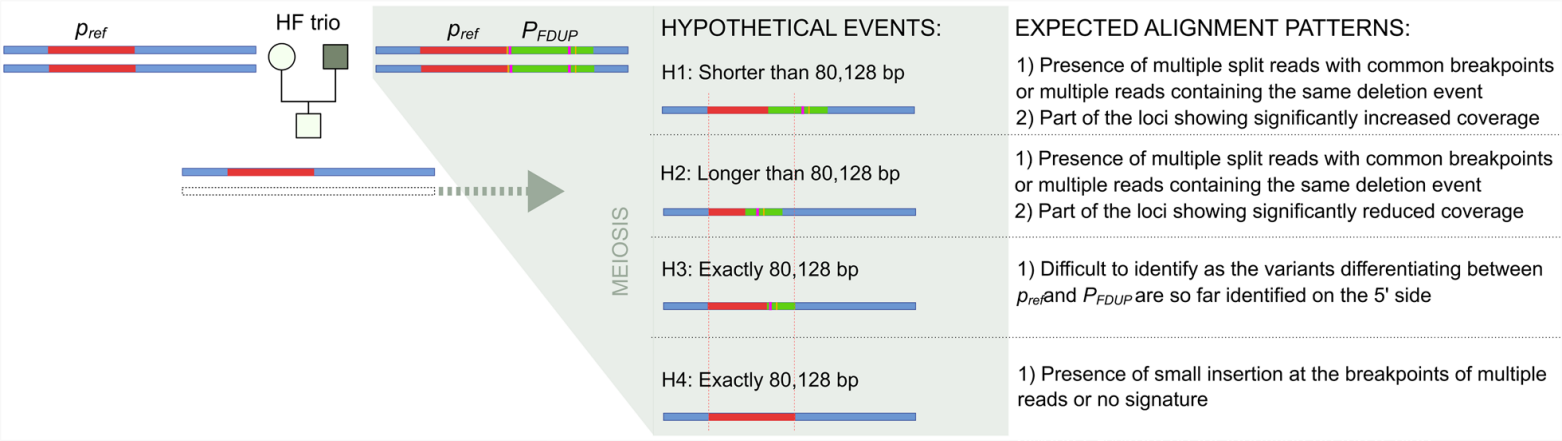
Overview of the hypotheses investigated in the HF trio. A schematic representation of the formulated hypotheses to explain the absence of the *P*_*F2D*_ variant in the offspring of the HF trio. A filled yellow rectangle and a filled pink rectangle, as depicted in the bar showing haplotype of the sire, represent InDel and SNP that are diagnostic markers in the *POLLED* test

For the HF trio, we hypothesize that part of the *P*_*F*_ variant’s duplication was deleted in the paternal germline. This deleted segment likely contains the causal motif for the polled phenotype. To explore this, we formulated four hypotheses (Fig. 1).

All hypotheses are based on the assumption that a region, encompassing both the SNP (*P*_*T-*>*A*_*)* and the 2 bp deletion (*P*_*F2D*_*,* TG), was deleted, leading to the absence of the *P*_*F*_ variant in diagnostic tests.

In Hypothesis 1 (H1), the deletion is shorter than 80,128 bp, leaving some duplicated sequence intact.

In Hypothesis 2 (H2), the deletion is longer than 80,128 bp, removing not only the entire duplication, but also a part of the wild-type sequence motifs. This leads to a de novo deletion rather than a duplication.

In Hypotheses 3 (H3) and 4 (H4), the deletion is exactly 80,128 bp. In H3, the deleted region includes sequences from both the wild-type and duplicated segments; in H4, only the duplicated sequence is deleted.

**Fig. 2:**
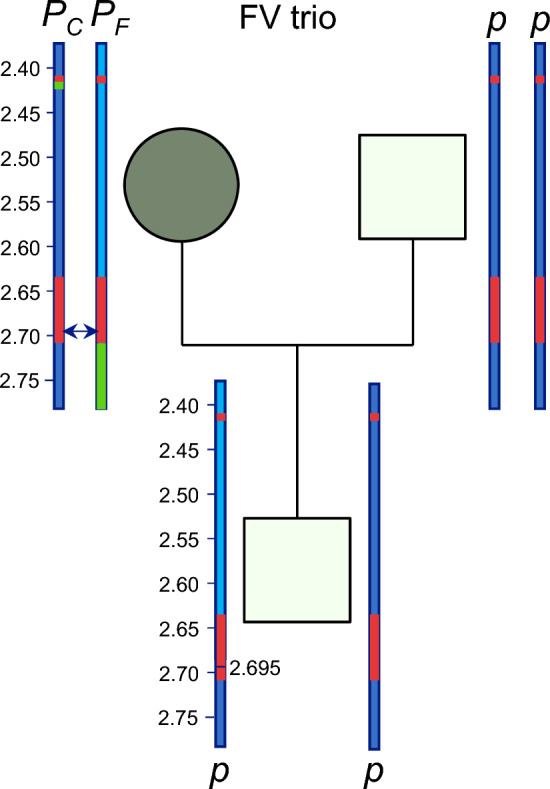
Overview of the hypothesis investigated in the Fleckvieh trio. A schematic representation of the haplotypes in the Fleckvieh trio. The *P*_*F*_ and *P*_*C*_ variants are shown in the trans phase of the maternal homologous chromosomes. The arrow between two maternal haplotypes indicates the most likely position of the recombination in the maternal germ cell

In the Fleckvieh trio, the dam carried both the *P*_*C*_ and *P*_*F*_ variants, each inherited from a different parent. As a result, the two variants were in the trans phase, located on opposite homologous chromosomes. We hypothesize that an allelic recombination event may have produced a recombinant haplotype in the offspring, in which the sequence up to the start of the duplicated region was derived from the chromosome carrying the *P*_*F*_ variant, while the sequence beyond that point originated from the chromosome carrying the *P*_*C*_ variant (Fig. 2).

This recombination would skip both the *P*_*C*_ and *P*_*F*_ variants, resulting in an offspring lacking polled variants.

We investigated each of these hypotheses by analysing whole genome sequencing (WGS) data generated using Oxford Nanopore Sequencing technology. The results obtained in this study shed light on the genomic composition of the *P*_*C*_ and *P*_*F*_ variants.

## Methods

### Sample information

**Fig. 3:**
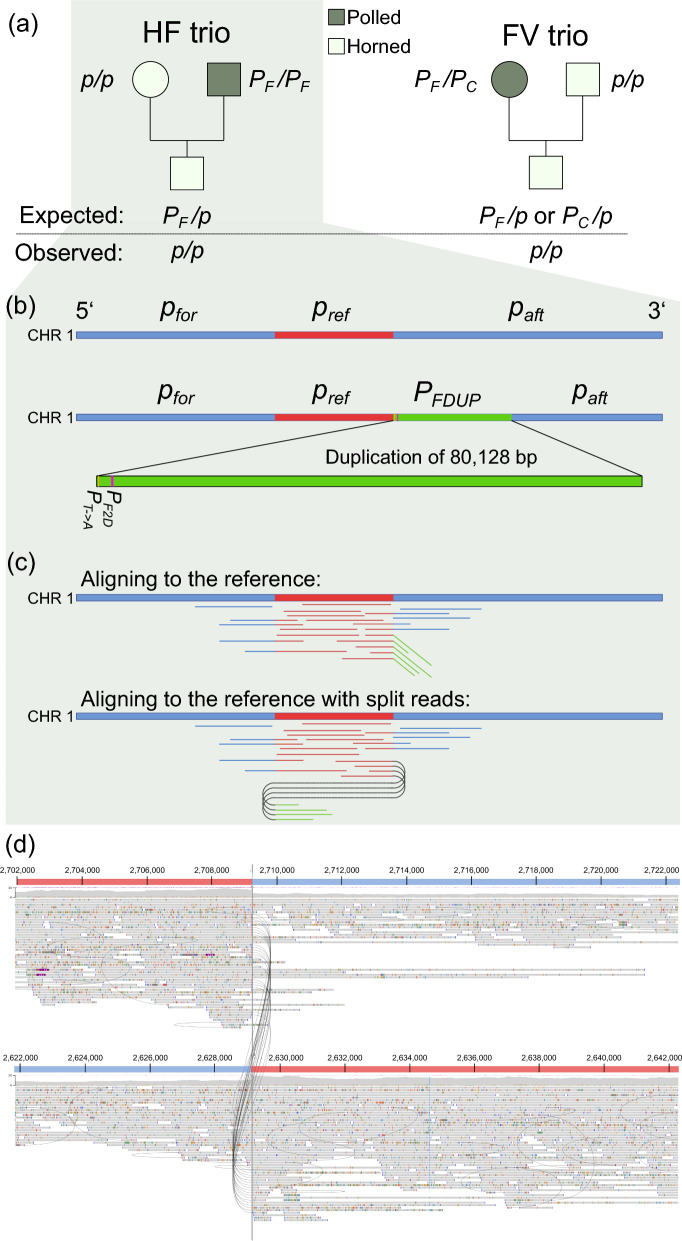
Overview of the inheritance patterns observed for the alleles of the *POLLED locus* in both the trios and genomic structure of the Friesian polled (*P*_*F*_) variant. **a** A schematic representation of the trios showing genotypes and allele inheritance at the POLLED locus. **b** A diagram of the wild-type locus and the Friesian polled (*P*_*F*_) variant. The *P*_*F*_ variant consists of a reference segment (*p*_*ref*_) and a duplicated segment (*P*_*FDUP*_). Two previously reported variants, *P*_*T*>*A*_ (a SNP) and *P*_*F2D*_ (a 2 bp InDel), are located on the 5′ side of the *P*_*FDUP*_ segment. **c** A Schematic illustration of how sequencing reads from a sample carrying the homozygous *P*_*F*_ variant align to the reference genome. **d** Representative screenshot showing split reads from the Holstein–Friesian (HF) sire homozygous for the *P*_*F*_ variant

The sire in the HF trio has over 13,000 registered offspring, of which only one examined here was declared to exhibit the horned phenotype. In the FV trio, the dam carried one copy of both the *P*_*C*_ and *P*_*F*_ variants, while the sire was tested as horned with the *p/p* genotype. The presence of the *P*_*F*_ variant in the FV population was expected, as this breed has introgressed with HF since the 1960s and thus carries a minor but significant genetic ancestry from the HF breed. In both trios, offspring were genotyped as *p/p* and exhibited regular horns fused to the frontal bone instead of the expected polled phenotypes and genotypes (Fig. 3).

### DNA extraction

The DNA was extracted from frozen samples of blood or semen (sires) immediately before the sequencing to get an increased proportion of long DNA fragments in the sequencing. The DNA extraction was carried out from 100 µl of blood using the QiaAmp Blood Mini Kit protocol (Qiagen, Hilden, Germany). Briefly, the resuspended blood was lysed and digested using proteinase K and RNase A, followed by binding to a column, washing, and elution through centrifugation.

### SNP genotyping and haplotyping for parentage verification and identification of recombination breakpoints

In the first step, both trios were analysed using SNP array genotypes to verify parentage and identify recombination breakpoints. Genotyping was performed using the Illumina BovineSNP50 BeadChip (Illumina, San Diego, USA) following the manufacturer’s protocol. All 54,157 markers were used to test the parentage in both trios. Subsequently, we unequivocally confirmed the expected pedigree in both trios, and for the FV trio, we even traced the pedigree to a much deeper level than the first-generation pedigree because our genotypic database included four FV grandparents. To investigate the recombination events, we inferred haplotypes and imputed missing genotypes using hidden Markov models as implemented in BEAGLE (v 5.0) [18, 19]. To improve the accuracy of the phasing, genotype and pedigree information of additional trios (n > 39,000) and pairs (n > 20,000) of both breeds genotyped with BovineSNP50K BeadChip, which were not otherwise included in the main analysis, were also included in this step of phasing.

### Diagnostic polled test to validate the presence of the *P*_*C*_ and *P*_*F*_ variants

The polled test was carried out according to the procedure and primers described in [9]. Briefly, the primer binding sites flanking the *P*_*C*_ variant InDel events (5′-TCAAGAAGGCGGCACTATCT-3′ and 5′-TGATAAACTGACCCTCTGCCTATA-3′) were PCR amplified (94 °C 30 s, 58 °C 60 s, 72 °C 60 s for 31 cycles). Next, PCR products were size-separated and visualized using 2% ethidium bromide-stained agarose gel electrophoresis. Genotyping of the *P*_*F*_ 80 kb duplication event was carried out using two primers flanking the variable site *P*_*F2D*_ (5′-GAAGTCGGTGGTCTGAAAGG-3′ and 5′-TGTTCTGTGTGGGTTTGAGG-3′). Next, PCR amplification (32 cycles, 94 °C 30 s, 59 °C 60 s, 72 °C 60 s) generated a RefSeq-related product (*p*_*ref*_); this product was obtained in all animals, be it horned or polled. Additionally, a second product related to the *P*_*F*_ variant, containing the two-base pair (TG) deletion, was only observed in the animals that carried one or two copies of the *P*_*F*_ variant (*P*_*FDUP*_) (Fig. 3). To discriminate between these two products related to the *P*_*F*_ variant, the ABI Prism 3130XL DNA sequencer was used. Animals carrying *P*_*F*_*/P*_*F*_ were distinguished from *P*_*F*_*/p* by using quantitative evaluation of the obtained signals. The animals carrying *P*_*F*_*/P*_*F*_ yielded signals of similar peak heights as the *P*_*F*_ and *p*_*ref*_ products, while heterozygous products (*P*_*F*_*/p*) resulted in signal intensities of approximately double height for the *p*_*ref*_ product when compared to the product specific to *P*_*F*_.

### Whole-genome long-read sequencing using Oxford Nanopore sequencing technology

To carry out long-read sequencing using the Oxford PromethION sequencer, 1 µg of unsheared DNA was end-repaired and A-tailed with NEBnext UltraII End-repair and A-tailing module (New England Biolabs, Ipswich, USA). The mixture was purified according to the manufacturer’s protocols with AmpureXP magnetic beads (Beckman Coulter, Brea, USA), ligated to 1D sequencing adapters (LSK114 kit, Oxford Nanopore Technologies (ONT), Oxford, UK), and again purified with AmpureXP beads. In the last Ampure cleanup step, instead of washing with 70% ethanol, a washing buffer supplied by Oxford Nanopore Technologies (LSK114 kit) was used. Approximately 200 ng of adapted DNA library was loaded onto a primed PromethION R10.4.1 M flow cell and sequenced for 72 h on a PromethION P24 sequencer.

### Bioinformatics pipeline to analyse WGS data

The samples were sequenced on a PromethION sequencer from Oxford Nanopore and were basecalled and demultiplexed during sequencing using Dorado (Dorado basecall server: v.7.3.11 + 0112dde09). After base calling, basic quality checks of the sequences were carried out using NanoPlot (v 1.41.6) [20]. Subsequently, the sequencing data were aligned to the cattle reference genome sequence (ARS-UCD1.3) using minimap2 (v 2.28) with secondary alignments suppressed [21, 22].

Investigation of the HF trio involved testing of the four hypotheses (Fig. 1). If hypotheses H1 or H2 were correct, we would have expected to detect multiple split reads with consistent breakpoints. Hypothesis H1 predicted a de novo duplication, whereas hypothesis H2 predicted a de novo deletion. It is important to note that, apart from a single SNP and a deletion, no additional variants have been identified that distinguish the *p*_*ref*_ and *P*_*FDUP*_ segments within the *P*_*F*_ variant. As a result, the testing of hypotheses H3 and H4 remains challenging. To identify genomic markers that could differentiate *p*_*ref*_ and *P*_*FDUP*_ segments as well as to test each of the above-mentioned hypotheses, a bioinformatics pipeline was developed for the comprehensive identification of genomic variants (SNPs and SVs) in the region of the *POLLED* locus (between 2.42 and 2.73 Mb on BTA1). Additionally, we visualized alignment patterns and assessed read depth. Small genomic variants (SNPs and short InDels) were identified using Clair3 (v 1.0.10) with the appropriate trained model for the basecaller. Structural variations (SVs) were detected using Sniffles2 (v 2.4), cuteSV (v 2.1.1), Dysgu (v 1.6.7), and SVIM (v 2.0.0) [23–27]. Moreover, SVs were also detected using a de novo genome assembly-based approach. For this purpose, Flye (v 2.9.5) [28] was used for de novo assembly of the trios, and SVIM-asm (v 1.3.0) [29] was used for the detection of SVs. Finally, all genomic variants were phased using LongPhase (v 1.7.3) [30].

To investigate the split-read alignments in the *POLLED* locus region and to identify recombination breakpoints in the offspring’s genome using the phased information of the parents’ genotypes, custom Python scripts were developed with the pysam library (https://github.com/pysam-developers/pysam) for efficient manipulation of the alignment data. The entire bioinformatics pipeline was implemented in the Nextflow workflow management system [31], which is available on GitHub at https://github.com/Popgen48/gvdlr/tree/main. To visualize alignment patterns in the BAM files, we used JBROWSE2 [32] and IGV [33].

### Primer design and validation of candidate variants

By examining the *POLLED* locus in both trios, we searched for short variants that would be specific to each trio. We found these putative variants: (a) a 1 bp deletion (*p*_*ref1D*_) and a SNP with transition (*p*_*refT-*>*C*_) in the HF trio, (b) a SNP with transition (*P*_*G-*>*A*_) in the Fleckvieh trio. For additional sequencing of the regions containing the mentioned three variants, we designed primers with Primer3Plus [34]. We also sequenced both ends of the duplicated segment *P*_*FDUP*_. Details about PCR products are explained in Table 1. The PCR amplification was performed with 35 cycles of 30 s at 94 °C, 60 s at 59 °C, and 60 s at 72 °C. PCR products of the trios were converted to barcoded nanopore sequencing libraries (SQK-NBD114.96) and sequenced on a PromethION R10.4.1 flowcell for approximately 2 h. The resulting fastq files were aligned on the cattle reference genome sequence (ARS-UCD1.3) using minimap2 (v 2.28) with secondary alignments suppressed. The BAM files were visualized using JBROWSE2.

**Table 1:** Information about PCR primers and their products used to validate candidate variants. In the HF trio, these variants were used to test the hypotheses H1–H4

Target	Location (in bp)	Trio	Primers	Product length	Annealing temperature
*P*_*G-*>*A*_	2,694,883	FV	5′-GGAACTGGAACTACTGAAGTCCT-3′	2943 bp	59 °C
			5′-TGTTTATCCCACAGCTGTTGGA-3′		
*P*_*T-*>*C*_	2,684,110	HF	5′-TGCATTGCTGTAGTCTGTTTGC-3′	1424 bp	58 °C
			5′-GGTAAGAGGTGCAGCCATGT-3′		
*p*_*ref1D*_	2,682,320	HF	5′-TATGTGCAGTGTGAGGCAGG-3′	1281 bp	65 °C
			5′-ATGAATCAGCCATGGGTGCA-3′		
*P*_*FDUP*_ left-end	2,629,116	HF	5′-GGGATCAGTCTTGGGCCCTA-3′	1268 bp	59 °C
			5′-TGACTTGGCCACAGACATCC-3′		
*P*_*FDUP*_ right-end	2,709,243	HF	5′-ATGCAGCTGGTCTTTCCTCC-3′	1338 bp	59 °C
			5′-ACCTTTCTACCATGCGGCAA-3′		

In the FV trio, the variant was used to validate the recombination-breakpoint. The location refers to the position on chromosome 1 on ARS-UCD1.3 assembly

## Results

The ONT sequencing produced an average of 36.03Gbp across the trios with an average read length N50 of 9.70 Kbp. A detailed overview of the sequencing statistics and alignment statistics is provided in Table 2.

**Table 2:** Basic sequencing and alignment statistics of the HF and FV trio

Sample id	genotypes	Relationship	Total bases sequenced (in Gbp)	Total number of reads sequenced	Read length N50 (in bp)	Mean alignment coverage
HF964	*P*_*F*_* P*_*F*_	Sire	59.20	15,907,973	7,786	22.98 ×
HF963	*pp*	Dam	25.00	2,670,919	13,986	9.68 ×
HF962	*pp*	Offspring	59.28	20,506,948	3,957	22.50 ×
FV2831	*pp*	Sire	18.92	3,564,540	8,379	7.24 ×
FV2822	*P*_*F*_* P*_*C*_	Dam	31.34	4,180,434	10,001	12.01 ×
FV2823	*pp*	Offspring	22.46	2,528,157	14,093	8.80 ×

### Analysis of the HF trio

The parentage of the offspring was validated using 50 K SNP array genotypes, and the diagnostic *POLLED* test revealed that the sire was homozygous for the *P*_*F*_ variant. This variant referred to the entire duplicated segment (Fig. 3): the original segment, *p*_*ref*_ (between 2,629,113 and 2,709,240 bp on BTA1), and its tandem duplicate (*P*_*FDU*P_). The *POLLED* test revealed the absence of *P*_*F2D*_ deletion in the offspring, pointing towards a de novo deletion of at least a part of the *P*_*F*_ variant involving *P*_*FDUP*_. Concerning this deletion, four hypotheses were formulated (Fig. 1). Next, each of these four hypotheses was investigated based on the results of comprehensive variant calling and manual inspections of the entire region. Note that recombination breakpoints could not be detected using 50 K SNP genotypes because the sire was homozygous for the genotypes in the *POLLED* locus.

Using the Nextflow pipeline developed specifically for this study for comprehensive identification of genomic variants from the ONT sequences in the *POLLED* locus of BTA1, a total of two SVs and two SNPs were detected in the sire (Fig. 3 and see Additional file 1, Table S1). Expectedly, the sire carried the *P*_*F*_ variant (Fig. 3). Interestingly, the visualization of the breakpoints identified 2 split reads that suggested that a part of the variant may be duplicated again (See Additional file 2, Fig. S1).

In the offspring, the Nextflow pipeline identified a total of one SV, an insertion, and eight SNPs in sequences potentially containing segments of *p*_*ref*_ and *P*_*FDUP*_. The SV (see Additional file 1, Table S1) was identified solely by the Dysgu tool; however, it was classified as a low-quality variant, as it was detected on reads with a mapping quality of 1, indicating multiple read alignments. Notably, no other tools identified any SVs supported by two or more reads. Furthermore, our custom Python tool, designed to detect split reads, did not find any region with two or more split reads sharing common breakpoints. Additionally, no regions larger than 1 kbp showed significantly higher or lower coverage (with more than one split read with consistent breakpoints) compared to the average coverage of the region. These findings collectively suggest that the hypotheses H1 and H2 (Fig. 1) could be rejected.

To investigate hypotheses H3 and H4, it was important to differentiate between reads that originated from the *p*_*ref*_ and those originating from *P*_*FDUP*_. However, apart from two variants (one SNP and a 2 bp deletion, *P*_*F2D*_) detected at the very beginning of the duplicated regions, no other variant that could differentiate the duplicated segment (*P*_*FDUP*_) from the reference sequence (*p*_*ref*_) has been identified in previous studies [9, 10]. To assess whether any trio-specific short variant could be used for this purpose, extensive manual inspection of the region was carried out in the BAM files of the sire as well as the offspring. A 1 bp deletion (*p*_*ref1D*_, located on 2,682,320 bp) present in the alignment of both sire and offspring was identified as one such candidate (see Additional file 3, Fig. S2). The long reads containing reference and alternative alleles at the candidate’s 1-bp deletion and SNP suggested their localization in the duplicated segment. However, the sequencing of PCR products to validate *p*_*ref1D*_ and the de novo SNP suggested that these variants were false positives (see Additional file 4, Table S2). Thus, it is most likely that the genomic region showing the sequence composition of *p/p* in the offspring may have been the result of a recombination event that occurred anywhere in the *P*_*F*_ segments and deleted almost exactly 80,128 bp of the genomic segments covering either a small part of the 3′ region of *p*_*ref*_ and a large part of the 5′ region of *P*_*FDUP*_ segment or the entire *P*_*FDUP*_ segment (hypothesis H3 or H4 in Fig. 1). Since insertions, duplications or multiple split reads with common breakpoints were not detected in the bioinformatic and manual (window-by-window) examination of the entire 80,128 bp in the HF offspring and the offspring’s sequence was identical (absence of duplication, *P*_*F*_ variant) to that of animal with the *p/p* genotype (e.g. dam), this strongly suggests that the deletion in the paternal germ cell was exactly 80,128 bp long, supporting either H3 or H4.

### Analysis of the Fleckvieh trio

The parentage of the offspring was validated using 50 K SNP array genotypes, and the diagnostic *POLLED* test revealed that the dam carried both *POLLED* alleles, *P*_*C*_ and *P*_*F*_. The Nextflow pipeline identified a total of two SVs and 179 SNPs in the combined region of the *P*_*C*_ and *P*_*F*_ variants. These two SVs were *P*_*C*_ and *P*_*F*_, confirming the results of the diagnostic *POLLED* test. Among these SVs, only *P*_*C*_ was detected by all the tools implemented in the workflow, while the *P*_*F*_ variant was not detected by Sniffles2, one of the most widely used tools for detecting SVs, and it was detected as having low quality by cuteSV. These variants were also visually confirmed using JBROWSE2 (see Additional file 5, Fig. S3 and Additional file 6, Fig. S4).

In the offspring, the Nextflow pipeline identified a total of three SVs and 810 SNPs in the combined region of the *P*_*C*_ and *P*_*F*_ variants. None of the identified SVs corresponded to the polled variants, *P*_*C*_ and *P*_*F*_, confirming the results of genotyping (*pp*). The higher number of SNPs was likely due to the higher coverage (than the dam); the sire also had high coverage and 910 SNPs.

To test the hypothesis of recombination (Fig. 2), we analysed the phased SNPs in the offspring. The analysis revealed that one of the maternal haplotypes was recombinant, showing a switch from one parental chromosome carrying the *P*_*F*_ variant to the other carrying the *P*_*C*_ variant. This recombination event was localized to position 2,694,883 bp on BTA1 (Fig. 2). This resulted in the skipping of both polled variants, *P*_*C*_ and *P*_*F,*_ in one of the haplotypes of the offspring. It should be noted that although the sequencing of PCR products did not validate the SNP reported by Clair3 at this position, the haplotype switching was obvious from this position onwards (see Additional file 7, Table S3). This was also supported by the recombination inferred using 50 K SNP genotypes of the trio (see Additional file 8, Table S4). Together, these genotyping data support the hypothesis of a recombination event occurring just before the start of the *P*_*FDUP*_ segments.

## Discussion

In this study, we conducted a detailed analysis of WGS data from the HF and FV trios, whose offspring exhibited unexpected inheritance patterns of the *P*_*C*_ and *P*_*F*_ variants. The results revealed that in one parent of each trio (both homozygous for the *POLLED* variants), meiotic recombination and crossover events led to the skipping of these variants, thereby reconstituting ancestral sequence motifs in the horned offspring. Furthermore, our findings suggest that the underlying mechanisms responsible for the regeneration of these ancestral variants may differ between the two trios. As the degree to which each *POLLED* variant suppresses horn growth varies, our findings also offer important insights linking the structural composition to functional aspects of the *POLLED* locus.

### The *P*_***F***_ variant suppresses horn growth better than the *P*_***C***_ variant

There is growing evidence that different *POLLED* variants in a heterozygous state suppress horn growth to varying degrees. For example, [35] found that almost every heterozygous *P*_*C*_*/p* Fleckvieh (FV) animal forms some kind of scurs, ranging from a minimal keratin crust to a horn fused to the frontal bone. The author concluded that the visibility and strength of the scurs depend on sex, age, and polygenic background of the FV animals. Later, we compared the frequency of scurs in heterozygous *P*_*F*_*/p* and *P*_*C*_*/p* animals and confirmed that *P*_*F*_ suppresses the development of horns significantly better than *P*_*C*_ and therefore, resulting in fewer animals exhibiting scurs and, when scurs occur, they are on average less pronounced [36]. Furthermore, there is evidence that the *P*_*M*_ variant completely suppresses the development of horns in heterozygous (*P*_*M*_*/p*) Mongolian Turano cattle and Mongolian yaks [11]. In our sample of 58 hornless Mongolian yaks and 40 hornless Mongolian Turano cattle, only 16 (30%) yaks and 5 (12.5%) Turano cattle were homozygous *P*_*M*_*/P*_*M,*_ and all others were heterozygous *P*_*M*_*/p*, but all were smooth-polled. After this observation, we tried to collect samples of Turano cattle and yaks with scurs. However, we found that breeders in Mongolia and China had never observed scurs in their animals and informed us that the animals were either horned or smooth-polled. This supports our observation that the *P*_*M*_ variant embedded in a large *P*_*F*_ duplication could be most effective at suppressing the development of horns in heterozygous yak and cattle.

### Indication of a non-allelic homologous recombination event in the paternal gamete of the HF trio

In a previous study [10], we used recombination within the *POLLED* locus to prove that segmental duplication *p*_*ref*_*-P*_*FDUP*_ is the only remaining causal candidate for the polledness of the Friesian origin. The back-mutation of *P*_*F*_ in the offspring of the HF trio investigated in this study offers the opportunity for additional confirmation of the *P*_*F*_ segmental duplication as the causal variant. The HF trio was analysed to decipher the genomic composition of the region containing the *P*_*F*_ variant. Specifically, after observing the missing *P*_*F2D*_ deletion in the offspring of a HF trio investigated here, our most parsimonious explanation would be that a portion of *P*_*F*_ in the paternal germ cell that led to the offspring must have been deleted. The deleted portion includes *P*_*F2D*_ and SNP *P*_*T-*>*A*_ at the transition from *p*_*ref*_ to *P*_*FDUP*_ (Fig. 1), but we assumed that some remaining parts of *P*_*FDUP*_ and *p*_*ref*_ should still be present in the paternal haplotype of HF offspring, i.e., one of the hypotheses H1 to H3 should apply. This would suggest that the part removed de novo in the *P*_*FDUP*_ segment caused the polledness in cattle. Surprisingly, our analysis identified neither deletion nor duplication in the entire segment in the offspring. Our observations ruled out hypotheses H1 and H2. The near-perfect reconstruction of the entire *p*_*ref*_ segment could be achieved by a deletion of exactly 80,128 bp and then fusion of the remaining parts of the *p*_*ref*_ and *P*_*FDUP*_ segments back to the wild-type sequence (H3 or H4, Fig. 1). It should also be considered that the wild-type variant created in such an arrangement is indistinguishable from the structural arrangement, in which the complete *P*_*FDUP*_ sequence is deleted (H4, Fig. 1). This is because *P*_*FDUP*_ is almost an exact duplicate of *p*_*ref*_, except for the two variants (*P*_*F2D*_ and SNP *P*_*T-*>*A*_) that are located at the beginning of the *P*_*FDUP*_ segment and not present in HF offspring. Therefore, any deletion of 80,128 bp in the *P*_*F*_ gamete, starting at any position between g.2,629,153 and g.2,709,243 will reconstruct the wild-type sequence. The only way to distinguish between H3 and H4 is to identify and confirm novel variants that are present in *P*_*FDUP*_ but not in the *p*_*ref*_ segment of the HF sire or vice versa. Our analysis did not identify any such high-quality candidate variant. Nevertheless, these results confirm that the polled phenotype in cattle requires duplication of either part of the *p*_*ref*_ genomic segment (as observed in the *P*_*M*_ variant) or of the entire *p*_*ref*_ genomic segment.

Segmental duplications (SDs) are duplicated blocks of genomic DNA typically ranging in size from 1–200 kb with a sequence identity of over 90% [37]. Thus, the duplicated blocks *p*_*ref*_ and *P*_*FDUP*_ can be classified by definition as long and almost perfect SDs. Typically, SDs and their flanking repeats are the sites of non-allelic homologous recombination (NAHR) [38]. The direction, orientation, as well as the chromosomal localization (intra-chromosomal or inter-chromosomal) of the duplicated segments involved in NAHR, determine the types of SVs that may arise [38–40]. Meiotic recombination is initiated by DNA double-strand breaks (DSBs). In the case of allelic homologous recombination (AHR), the damaged sequence around the DSB is usually repaired by replacement with the allelic sequences of the sister chromatids as a template. In most cases, this recombination process is highly faithful and ensures accurate distribution of alleles and chromosomes as long as the DSB occurs in the unique regions of the genome. In the case of a DSB within the SD regions, the replication machinery may find the segment’s paralogs instead of the actual allelic segment for repair, resulting in NAHR [41]. All types of NAHR (intra-chromatid, inter-sister chromatid, or interhomologue) can generate a deletion or other recurrent genomic rearrangements. Based on the results, it is likely that during the DSB repair in the region of the *P*_*F*_ variant, the replication machinery found the paralog instead of the allelic segment, thus resulting in a hybrid SD [40]. As the repeat sequences are highly similar, the process skipped a genomic region equal in size to the duplicated segment, producing a perfect deletion of one of the two segments. It is noteworthy that the HF sire has more than 13,000 offspring, so it is plausible that a gamete carrying such a rare recombination event gave rise to the offspring investigated here.

### Indication of allelic homologous recombination in the maternal gamete of the FV trio

The polled variants *P*_*F*_ and *P*_*C*_ are the result of two independent mutations in different subpopulations of the bovine species. Therefore, they segregate on different haplotype backgrounds. In some breeds, such as Jersey, Fleckvieh, and Holstein, both variants are inherited independently in different lines, and, in rare cases, animals inherit *P*_*C*_ from one parent and *P*_*F*_ from another. These animals are homozygous for *POLLED*, denoted as *PP*, but heterozygous for the Celtic and Frisian mutations, which are 200 kbp apart. The dam of the Fleckvieh trio is such an animal with *P*_*C*_ and *P*_*F*_ alleles in the trans phase. According to [42], an average of 1.085 Mb corresponds to 1.0 cM in female cattle. Therefore, on average, we expect two out of 1,000 oocytes to show recombination between the *P*_*C*_ and *P*_*F*_ alleles. It is expected that this proportion is even lower for the *POLLED* locus, as all cattle autosomal chromosomes are acrocentric [43] and regions near the centromere show the lowest recombination rate [43–45]. According to the results obtained from the analysis of the long-read sequences of FV-Trios (Fig. 2), the following scenario seems to be most probable: during prophase of the first meiotic division in the dam of the FV trio, a DSB was introduced in one of the sister chromatids carrying the *P*_*F*_ variant. This most likely occurred closer to the distal end of the *p*_*ref*_ segment, ~ 14 kbp upstream from the start of the *P*_*FDUP*_ segment. This DSB was repaired by crossover with the allelic position of a non-sister chromatid containing the *P*_*C*_ variant proximal to the DSB and the *p*_*ref*_ segment distal to the DSB. The crossover fused the proximal portion of the *P*_*F*_ chromatid with the distal portion of the *P*_*C*_ chromatid, resulting in the skipping of both *POLLED* variants and the reconstruction of the wild-type haplotype.

### Practical implications of the findings

The results of our study point toward some interesting observations and raise intriguing possibilities about the outcomes of recombination events. First, the recombination breakpoint inferred in the gametes of the dam is within a 1-kb distance of the *P*_*M*_ variant. Next, in both the trios, the resulting hybrid SD has a similar composition (i.e., the wild-type arrangement was reconstructed), but was generated by different types of recombination. In theory, it is equally likely that the crossover event in the FV dam fuses the *P*_*C*_ and *P*_*F*_ variants into a *cis*-haplotype, i.e., both located on the same chromosome. In this case, the offspring with *P*_*C*_ and *P*_*F*_ in a *cis*-arrangement would be phenotypically polled and would most likely not be observed as something unexpected and therefore falsely declared as homozygous polled, *PP*. Similarly, the recombination within the SD of the *P*_*F*_ variant could lead to diverse rearrangements that could result in unexpected phenotypes and genotypes in offspring, even when sires or dams are tested as genetically homozygous polled. Therefore, it is important that any deviation from the expected results at the large *POLLED* locus is critically analysed. Possibly, some of these cases can further narrow down the sequence motif that is essential for polledness in cattle.

Our results also indicated that comprehensive detection of variants using the ONT sequences should involve the use of multiple tools employing a variety of approaches. For instance, in the FV dam, while the *P*_*C*_ variant was identified confidently by all the tools implemented in the workflow, the *P*_*F*_ variant was missed by Sniffles2 and detected as a low-quality variant by cuteSV. Furthermore, comprehensive detection of short variants such as InDels and SNPs with high confidence is difficult using long-read sequencing technology like ONT due to their relatively high error rates. Therefore, our data suggest that the short variants identified using ONT sequencing should be thoroughly validated even after applying a variety of algorithms.

## Conclusions

Here, we comprehensively investigated the genomic composition of Friesian and Celtic polled variants by using 50 K genotyping data and long-read sequencing data generated from Holstein–Friesian and Fleckvieh trios. Our data support the hypothesis that the 80-kbp duplication is the genetic variant responsible for the polled phenotype of Friesian origin. We also showed that different genomic arrangements, because of allelic homologous recombination and non-allelic homologous recombination in the *POLLED* locus, can lead to the emergence of de novo ancestral horn phenotypes. Such genomic arrangements can complicate phenotype prediction in offspring, even when sires or dams are tested as genetically homozygous polled for the *P*_*F*_ allele using SNP-based diagnostics. Therefore, it is important for the large *POLLED* locus that any deviation from the expected result is critically analysed. Some of these cases may further narrow down the sequence motif essential for polledness in cattle.

## Supplementary Information

Additional file 1.

Additional file 2.

Additional file 3.

Additional file 4.

Additional file 5.

Additional file 6.

Additional file 7.

Additional file 8.

## Data Availability

The WGS data from both trios have been deposited in the European Nucleotide Archive (ENA) under project ID: PRJEB98370. All the scripts and workflows used for the analysis can be accessed on the GitHub page: https://github.com/Popgen48/gvdlr.
