# Supplementary material for: Recombination events restored the functional horned haplotypes in the offspring of polled parents
Source: Genet Sel Evol. 2025 Oct 31;57:65. doi: 10.1186/s12711-025-01009-6 (PMC12579413; doi:10.1186/s12711-025-01009-6)
Supplement: Supplementary file 2 — Additional file 2. [file 12711_2025_1009_MOESM2_ESM.docx]

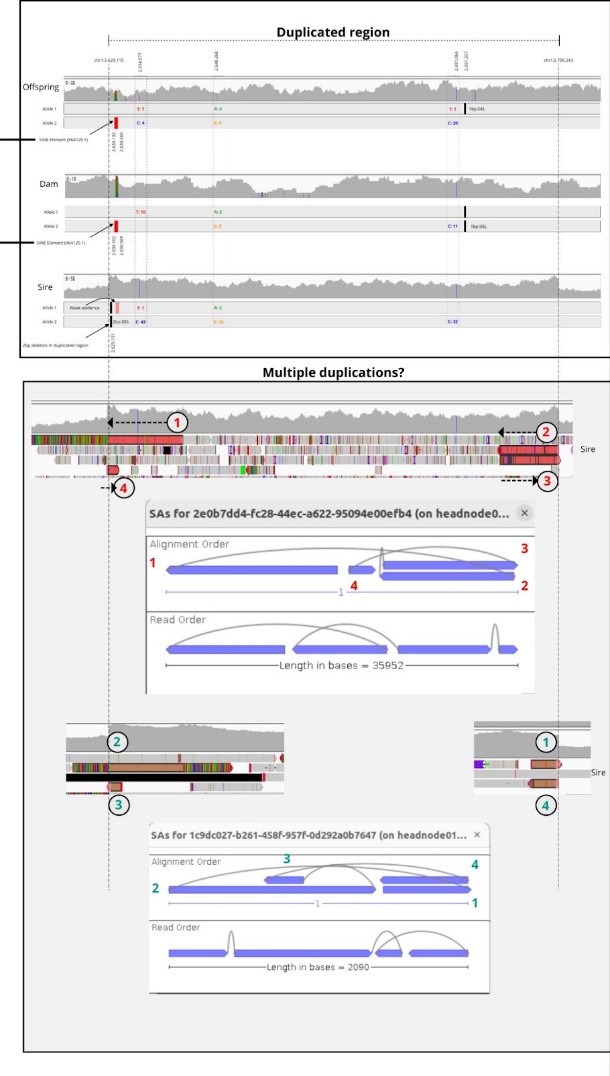


**Figure S1. Nanopore reads from the sire of the HF trio indicate complex duplication of the PF variant**

Two representative Nanopore reads from the sire of the HF trio display split alignments in a genome browser (IGV), with read fragments mapping to the start and end of the duplicated region. The dotted line marks the duplicated genomic segment. The alignment order and read order below show that the reads break at the boundaries of the duplicated region and continue aligning to other positions within the same genomic locus, suggesting the presence of multiple and potentially rearranged copies of the *P_F_* variant, indicative of a complex duplication.
