# Supplementary material for: Recombination events restored the functional horned haplotypes in the offspring of polled parents
Source: Genet Sel Evol. 2025 Oct 31;57:65. doi: 10.1186/s12711-025-01009-6 (PMC12579413; doi:10.1186/s12711-025-01009-6)
Supplement: Supplementary file 3 — Additional file 3. [file 12711_2025_1009_MOESM3_ESM.docx]

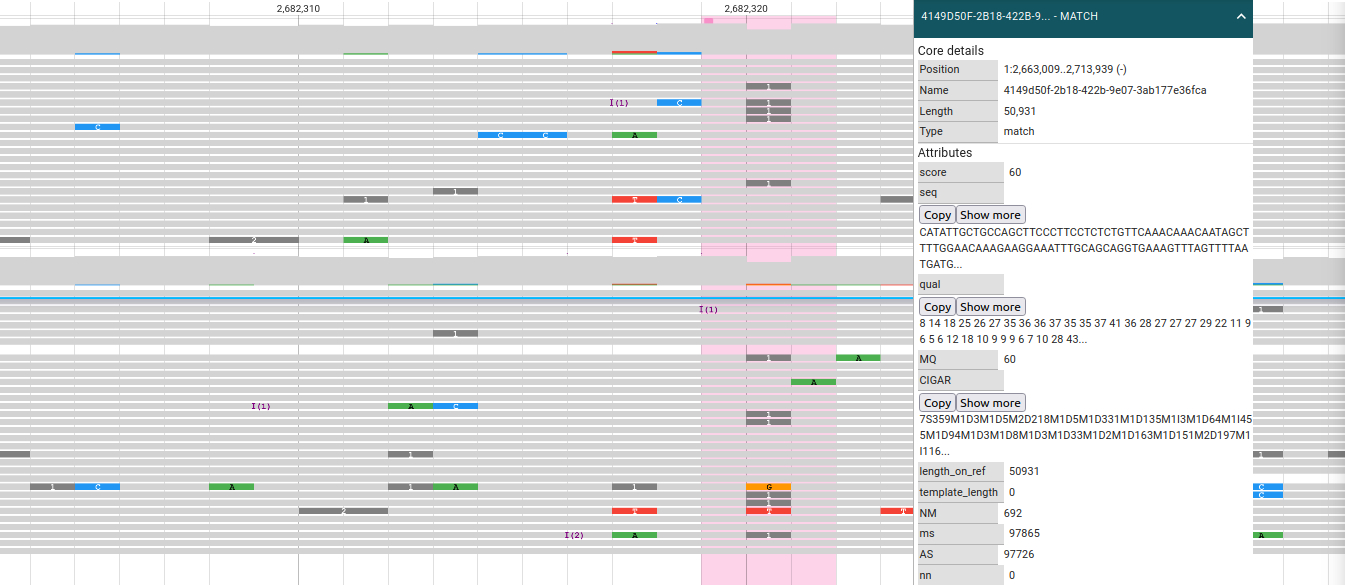


**Figure S2.** Screenshot from JBrowse showing a 1 bp deletion (highlighted in pink) identified in both the sire and offspring of the HF trio. This deletion is inferred to be present on the p_ref_ haplotype, based on the alignment of two reads in the sire that span the breakpoint without exhibiting the deletion or requiring split alignment. Detailed mapping information for one of these reads is shown.
