# Supplementary material for: Recombination events restored the functional horned haplotypes in the offspring of polled parents
Source: Genet Sel Evol. 2025 Oct 31;57:65. doi: 10.1186/s12711-025-01009-6 (PMC12579413; doi:10.1186/s12711-025-01009-6)
Supplement: Supplementary file 4 — Additional file 4. [file 12711_2025_1009_MOESM4_ESM.docx]

**Table S2**. The result of very high coverage sequencing of PCR products using ONT technology to validate the candidate variants.

| **Variant** | **Trio** | **Expected pattern** | **Observed pattern** | | | **conclusion** |  |
| --- | --- | --- | --- | --- | --- | --- | --- |
|  |  |  | **sire** | **dam** | **offspring** |  |  |
| *P_G->A_* | FV | only dam as heterozygous | ~45,000 reads aligned, of which ~0.6% showed this SNP | ~35,000 reads aligned of which ~0.6% showed this SNP | ~11,000 reads aligned of which ~0.6% showed this SNP | False positive |  |
|  |  |  |  |  |  |  |  |
| *P_T->C_* | HF | *de-novo* in offspring | ~4098 reads aligned, of which 4% showed this SNP | ~7708 reads aligned of which 3% showed this SNP | ~11,140 reads aligned of which 4% showed this SNP | False positive |  |
|  |  |  |  |  |  |  |  |
| *p_ref1D_* | HF | Present only in sire and offspring | ~1245 reads aligned, of which 9% showed this deletion | ~2300 reads aligned, of which ~8% showed this deletion | ~4155 reads aligned, of which ~7% showed this deletion | False positive |  |
