# Supplementary material for: Recombination events restored the functional horned haplotypes in the offspring of polled parents
Source: Genet Sel Evol. 2025 Oct 31;57:65. doi: 10.1186/s12711-025-01009-6 (PMC12579413; doi:10.1186/s12711-025-01009-6)
Supplement: Supplementary file 5 — Additional file 5. [file 12711_2025_1009_MOESM5_ESM.docx]

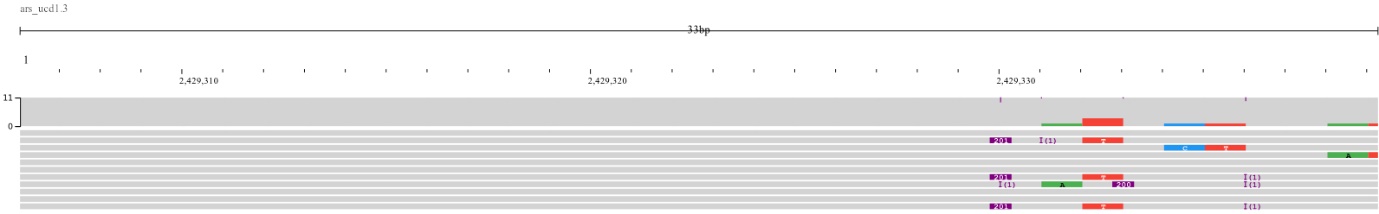


**Fig. S3.** The *P_C_* variant is visually confirmed in the dam of FL trio; It is supported by four reads and highlighted in pink within the boxed region.
