# Supplementary material for: Recombination events restored the functional horned haplotypes in the offspring of polled parents
Source: Genet Sel Evol. 2025 Oct 31;57:65. doi: 10.1186/s12711-025-01009-6 (PMC12579413; doi:10.1186/s12711-025-01009-6)
Supplement: Supplementary file 6 — Additional file 6. [file 12711_2025_1009_MOESM6_ESM.docx]

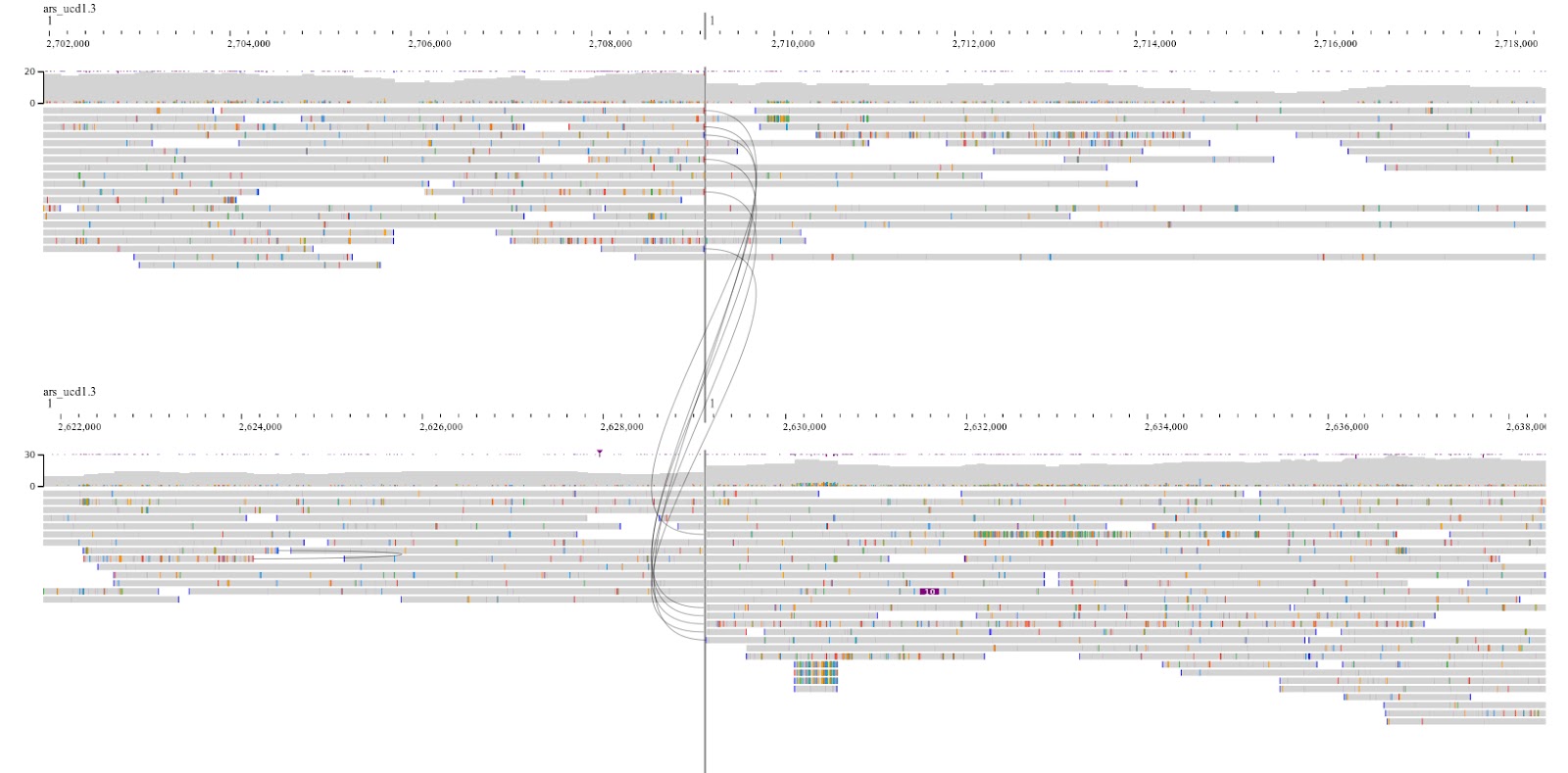


**Figure S4.** The *P_F_* variant is visually confirmed in the dam of FL trio; the split-read alignments are seen in both the panels, referring to the tandem duplication.
