# Supplementary material for: Recombination events restored the functional horned haplotypes in the offspring of polled parents
Source: Genet Sel Evol. 2025 Oct 31;57:65. doi: 10.1186/s12711-025-01009-6 (PMC12579413; doi:10.1186/s12711-025-01009-6)
Supplement: Supplementary file 8 — Additional file 8. [file 12711_2025_1009_MOESM8_ESM.docx]

AnimalLID Nr 000000000100000000020000000003000000000400000000050000000006000000000700000000080000000009

AnimalLID Nr 123456789012345678901234567890123456789012345678901234567890123456789012345678901234567890

FV2822K M GGCAGAAGGAAGGACGAGCCAGGGAGAGAGCAAAGACAAGGAAGACAGAGCAAAAGGAAAAGGAGGAGAGGGAAGGCGGGACGGAGAGC

FV2822K P AGCAGGGAGAAGGACGACCCGGGAAGAGGGCGAAGACAAGGAGGGCGAAGCGGCCGGGGAAGGAGGAGAAGGGCGGAGGAAAGGAGAAC

FV2823K M GGCAGGGGGGAGGACGACAAGAGAAAAGAGCAGCAGGGGAAGGGGCGAGGCAGCCGGGGCAGAAGAGGAAGGGCGGAGGAAAGGGGAGA

FV2823K P AGCAGGGAGAAGGACGACCCGGGAAGAGGGCGAAGACAAGGAAGACAGAGCAAAAGGAAAAGGAGGAGAGGGAAGGCGGGACGGAGAGC

FV2831 M AACAGAGGGGGAGACGACCCGGGACAAGAGCAACAGGGGAAGGGGCGAGGCAGCCGGGGCAGAAGAGGAAGGGCGGAGGAAAGGGGAGA

FV2831 P GGCAGGGGGGAGGACGACAAG**A**GAAAAGAGCA**G**CAGGGGAAGGGGCGAGGCAGCCGGGGCAGAAGAGGAAGGGCGGAGGAAAGGGGAGA

**Chr Pos MarkerID a1 a2**

1 776231 Hapmap43437-BTA-101873 A G

1 907810 ARS-BFGL-NGS-16466 A G

1 1032564 Hapmap34944-BES1_Contig627_1906 A C

1 1073496 BTA-07251-no-rs A G

1 1110393 ARS-BFGL-NGS-98142 A G

1 1150763 Hapmap53946-rs29015852 A G

1 1167048 BFGL-NGS-114208 A G

1 1204825 ARS-BFGL-NGS-66449 A G

1 1566539 ARS-BFGL-BAC-32770 A G

1 1604940 ARS-BFGL-NGS-65067 A G

1 1650662 ARS-BFGL-BAC-32722 A G

1 1671886 ARS-BFGL-BAC-34682 A G

1 1695632 ARS-BFGL-NGS-3964 A G

1 1730547 ARS-BFGL-NGS-98203 A C

1 1760809 ARS-BFGL-NGS-59105 A C

1 1834781 ARS-BFGL-BAC-2376 A G

1 1909740 ARS-BFGL-BAC-31722 A G

1 1929664 BTA-49284-no-rs C G

1 1954528 ARS-BFGL-BAC-6557 A C

1 1984725 ARS-BFGL-BAC-7196 A C

1 2008865 ARS-BFGL-BAC-12931 A G

1 2032673 ARS-BFGL-BAC-649 A G

1 2058539 ARS-BFGL-BAC-14857 A G

1 2082139 Hapmap53766-ss46526150 A G

1 2107796 BFGL-NGS-115971 A C

1 2158691 ARS-BFGL-NGS-62826 A G

1 2235492 BTA-39394-no-rs A G

1 2265614 ARS-BFGL-NGS-109301 A G

1 2305128 ARS-BFGL-NGS-105306 A G

1 2327877 ARS-BFGL-NGS-87636 A G

1 2348424 Hapmap24070-BTA-123581 A C

1 2391777 ARS-BFGL-NGS-39992 A G

1 2459049 ARS-BFGL-BAC-4401 A G

1 2482685 ARS-BFGL-BAC-13059 A C

1 2505294 ARS-BFGL-BAC-14930 A G

1 2528158 ARS-BFGL-BAC-15552 A G

1 2551347 ARS-BFGL-BAC-15580 C G

1 2574098 ARS-BFGL-NGS-11433 A G

1 2617150 ARS-BFGL-NGS-25183 A G

1 2641517 ARS-BFGL-NGS-76349 A G

1 2673226 ARS-BFGL-BAC-16253 A G

**1 2703664 ARS-BFGL-BAC-7317 A G =42**

**1 2769164 ARS-BFGL-NGS-29653 A G =43**

1 2809407 ARS-BFGL-BAC-14982 A G

1 2848684 BTA-120704-no-rs A G

1 2893133 ARS-BFGL-BAC-6733 A C

1 2931328 ARS-BFGL-NGS-85297 A G

1 3010910 ARS-BFGL-NGS-16727 A G

1 3033433 BFGL-NGS-115671 A G

1 3114153 ARS-BFGL-NGS-71085 A G

1 3135408 ARS-BFGL-NGS-21352 A C

1 3182687 ARS-BFGL-NGS-103391 A G

1 3245141 ARS-BFGL-NGS-22858 A G

1 3271366 ARS-BFGL-NGS-17308 A C

1 3303269 ARS-BFGL-NGS-92033 A C

1 3365623 ARS-BFGL-NGS-89044 A G

1 3396953 ARS-BFGL-NGS-79093 A G

1 3434539 ARS-BFGL-NGS-58892 A G

1 3470316 ARS-BFGL-NGS-98287 A G

1 3492221 BTB-00001612 A C

1 3520298 Hapmap53592-rs29009975 A G

1 3560291 ARS-BFGL-NGS-15775 A G

1 3599891 ARS-BFGL-NGS-59192 A G

1 3621606 ARS-BFGL-NGS-101618 A G

1 3653664 BTB-00001299 A G

1 3687775 ARS-BFGL-NGS-37412 A G

1 3721639 ARS-BFGL-NGS-16784 A G

1 3790017 BTB-02081949 A G

1 3826765 BTB-00001074 A G

1 3859505 ARS-BFGL-NGS-43924 A G

1 3884274 BTA-26294-no-rs C G

1 3908027 ARS-BFGL-NGS-30999 A G

1 3958736 Hapmap57114-rs29012843 A G

1 3958867 ARS-USMARC-Parent-DQ381153-rs29012842 A C

1 4004958 ARS-BFGL-NGS-37505 A G

1 4052172 ARS-BFGL-NGS-107481 A G

1 4086403 ARS-BFGL-NGS-108686 A C

1 4113357 BTA-31687-no-rs A G

1 4142326 ARS-BFGL-NGS-36096 A G

1 4168130 BTA-32603-no-rs A G

1 4204146 ARS-BFGL-NGS-109014 A G

1 4250046 ARS-BFGL-NGS-69957 A C

1 4276065 ARS-BFGL-NGS-43005 A G

1 4309205 Hapmap59750-rs29024375 A G

1 4335252 BTB-00002335 A G

1 4357492 ARS-BFGL-NGS-107287 A G

1 4394936 Hapmap42514-BTA-33574 A G

1 4494007 ARS-BFGL-NGS-81011 A G

1 4547357 BTB-00003162 A C
